# Efficacy of Nivolumab in a Patient with Metastatic Renal Cell Carcinoma and End-Stage Renal Disease on Dialysis: Case Report and Literature Review

**DOI:** 10.1155/2018/1623957

**Published:** 2018-06-13

**Authors:** Jawaher Ansari, Muhammad Ali, Ashraf Farrag, Arwa M. Ali, Abdulaziz Alhamad

**Affiliations:** ^1^Department of Oncology, Prince Sultan Military Medical City, Riyadh, Saudi Arabia; ^2^Clinical Oncology Department, Assiut University, Asyut, Egypt; ^3^Medical Oncology Department, South Egypt Cancer Institute, Asyut, Egypt

## Abstract

Treatment of patients with metastatic renal cell carcinoma (mRCC) and end-stage renal disease (ESRD) on dialysis poses a therapeutic challenge, particularly as this patient group was excluded from the pivotal clinical trials. In addition, there is uncertainty regarding drug dosing/pharmacokinetics, lack of safety and efficacy data, and potential for increased toxicity when using targeted therapy or immunotherapy for the management of patients with mRCC on dialysis. Nivolumab, an anti-programmed death-1 immune checkpoint inhibitor antibody, is indicated for the treatment of patients with mRCC who have received prior antiangiogenic therapy. Given the above-mentioned uncertainties, clinicians may be reluctant to use nivolumab for this patient population, leading to potential denial of life-prolonging medications. We report the case of a 72-year-old gentleman with mRCC and ESRD on dialysis who received second-line nivolumab therapy and achieved an excellent symptomatic and radiological response, remaining progression-free for over 22 months. In addition, we have reviewed the pharmacokinetic data and published retrospective case studies to review the management options for patients with mRCC and ESRD on dialysis.

## 1. Introduction

Renal cell carcinoma (RCC) is the 13^th^ most common cancer worldwide, with around 338,000 cases diagnosed annually [1]. Recent advances and better understanding of the RCC tumour biology have led to the advent of several new targeted agents and immunotherapy drugs in the RCC armamentarium. Nivolumab is a fully human Immunoglobulin G4 (IgG4) programmed death-1 (PD-1) immune checkpoint inhibitor antibody that selectively blocks the interaction between PD-1, which is expressed on activated T cells, and its ligands PD-L1 and PD-L2, which are expressed on immune cells and tumour cells. In November 2015, the US Food and Drug Administration (FDA) approved nivolumab for use in patients with advanced RCC who have received prior antiangiogenic therapy. In April 2018, the US FDA approved the combination of nivolumab and ipilimumab, an anti-cytotoxic T-lymphocyte antigen-4 antibody, for the treatment of intermediate or poor risk, previously untreated advanced RCC based on the results of the phase III CheckMate 214 trial [2].

The true incidence of end-stage renal disease (ESRD) and dialysis in metastatic RCC (mRCC) patients remains unknown; however, the prevalence of RCC appears to be higher in patients with ESRD when compared to the general population [3]. Treatment of patients with mRCC and ESRD on dialysis poses therapeutic challenges due to a variety of reasons, such as the uncertainty regarding drug dosing/ pharmacokinetics, lack of safety and efficacy data, potential for increased toxicity, and coexisting comorbidities. This could potentially lead to undertreatment or denial of life-prolonging drugs to mRCC patients with ESRD undergoing dialysis. Patients with ESRD are often excluded from prospective clinical trials because of their altered pharmacokinetics and comorbidities.

There is very limited evidence regarding the efficacy or tolerability of nivolumab in patients with renal impairment or those on dialysis. The summary of product characteristics for nivolumab states that no dose adjustment is required for patients with mild or moderate renal impairment; however, there is no specific information regarding patients on dialysis. To date, no controlled clinical trials/studies have evaluated the efficacy and safety of nivolumab in patients with RCC having renal impairment or undergoing dialysis. A search of published literature (PubMed and EMBASE) from 2000 to present using the search terms, nivolumab/kidney/renal/dialysis, identified only two cases of metastatic RCC and one case of metastatic melanoma and ESRD on dialysis which received nivolumab [4–6]. Additionally, a detailed search of www.clinicaltrials.gov did not identify any planned, ongoing, or completed trials evaluating the efficacy and safety of nivolumab in patients with renal impairment or undergoing dialysis.

We report the case of an elderly gentleman with mRCC and ESRD on dialysis who received second-line nivolumab therapy despite poor performance status and multiple comorbidities. He had an excellent radiological and symptomatic response to nivolumab and remains progression-free 22 months from treatment initiation. In addition, we have reviewed the evidence for various treatment options in the management of patients with mRCC and ESRD on dialysis.

## 2. Case Presentation

This 72-year-old gentleman presented with a 6-week history of haematuria and underwent a computed tomography (CT) scan that revealed a left renal tumour suggestive of RCC. His comorbidities included type 2 diabetes mellitus and hypertension. He had no family history of any malignancy. He was a life-long nonsmoker and his Eastern Cooperative Oncology Group (ECOG) performance status was 1. He underwent left partial nephrectomy and histology revealed this to be a locally advanced clear cell RCC, Fuhrman grade 2, with involvement of 3 out of 20 lymph nodes (pT3A N1 M0). Postoperatively, he developed ESRD and was started on dialysis 3 times/week. Two years later, he developed a local recurrence in the left kidney and underwent left radical nephrectomy. Histopathology revealed a 5 cm, clear cell carcinoma, Fuhrman grade 2 with invasion of the perinephric fat and renal vessels. He remained on regular follow-up and unfortunately 2 years later he developed further disease progression with a renal bed recurrence along with multiple bone and lung metastases. He received high-dose palliative radiotherapy to the renal bed 40 Gray in 20 fractions followed by commencement of systemic treatment with dose-reduced pazopanib. The dose of pazopanib was reduced to 200 mg daily due to his poor ECOG performance status of 3 and ongoing renal dialysis. Unfortunately, follow-up CT scan 3 months later showed significant disease progression in the renal bed, bone, and lung metastases. He also developed significant pain (score 8 out of 10) over his left loin secondary to the renal bed metastatic deposit.

**Figure 1:**
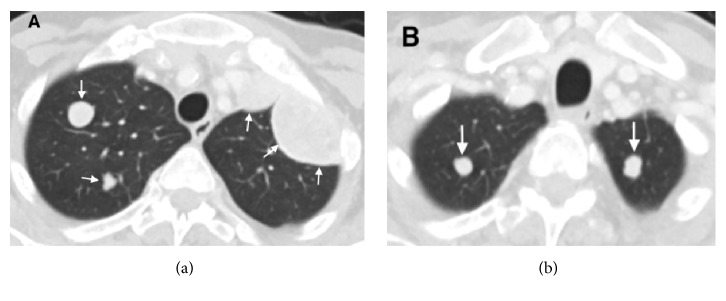
Axial contrast-enhanced computed tomography (CT) scan of chest showing bilateral multiple lung metastases (white arrows) prior to nivolumab treatment.

**Figure 2:**
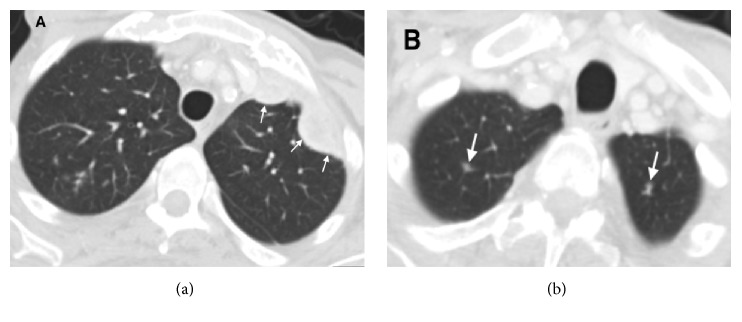
Axial contrast-enhanced corresponding CT scan images after 12 weeks of nivolumab treatment showing a partial response with reduction in size of multiple bilateral lung metastases (white arrows).

He was started on nivolumab 3 mg/kg initially and later switched to 240 mg flat dose intravenously every 2 weeks. He tolerated the treatment extremely well with no grade 2-4 toxicities. Clinically, there was a significant improvement in his pain control with a reduction in his pain score from 8/10 to 3/10 and his ECOG performance status improved to 2. Follow-up CT scans showed a partial response as per response evaluation criteria in solid tumours (RECIST) version 1.1. There was a significant reduction in the size of lung and renal bed metastases (Figures 1 and 2). He remains on nivolumab 22 months from initiation of treatment with serial imaging showing ongoing response.

## 3. Discussion

For patients with mRCC, treatment options in the first-line setting include vascular endothelial growth factor receptor (VEGFR) tyrosine kinase inhibitors (TKIs): sunitinib and pazopanib; a monoclonal anti-vascular endothelial growth factor (VEGF) antibody, bevacizumab in combination with interferon-*α*; mammalian target of rapamycin (mTOR) inhibitor: temsirolimus; and immunotherapy with high-dose interleukin-2. More recently, the immunotherapy combination of nivolumab and ipilimumab has been approved for patients with intermediate or poor risk advanced RCC [2]. Significant advances have been made in the second-line treatment of mRCC and the options now include TKIs—axitinib and cabozantinib; an anti-PD1 monoclonal antibody—nivolumab; and an mTOR inhibitor, everolimus, either alone or in combination with lenvatinib, a TKI. However, patients with severe renal impairment or those on dialysis were excluded from the pivotal trials of these agents, posing a therapeutic dilemma for day-to-day clinical practice.

With regard to VEGFR TKIs and mTOR inhibitors, there is some evidence to suggest that mRCC patients on dialysis treated with these agents have similar outcomes in terms of both efficacy and safety, compared to mRCC patients with normal or minimally impaired kidney function [7–10]. On the contrary, some reports have shown a higher incidence of adverse events with VEGFR TKIs in mRCC patients on dialysis despite initiation of therapy at reduced doses [11]. However, this evidence is based on case reports and retrospective case series confounded by limited patient numbers and potential reporting bias [12].

Currently, nivolumab is the only checkpoint inhibitor approved for the second-line treatment of mRCC patients after failure of TKIs. The pivotal CheckMate-025 phase 3 trial randomized 821 mRCC patients, who had received prior antiangiogenic therapy, to either nivolumab or everolimus [13]. The results demonstrated a higher response rate (25% versus 5%; odds ratio, 5.98 [95% CI, 3.68 to 9.72]; *P* < 0.001) and improved median overall survival (25 months versus 19.6 months; HR: 0.73 [98.5% CI, 0.57 to 0.93]; *P* = 0.002) for nivolumab-treated patients. The inclusion criteria for CheckMate-025 trial specified a serum creatinine level ≤ 1.5 x ULN or creatinine clearance ≥ 40 mL/min, thereby excluding patients with moderate-to-severe renal impairment.

In a population-based pharmacokinetics model that assessed covariate effects on nivolumab concentrations in 1,895 patients who received nivolumab in 11 clinical trials, there was no clinically important difference in the clearance and exposure of nivolumab between patients with renal impairment and those with normal renal function [14]. The absence of a relationship between estimated glomerular filtration rate (eGFR) and nivolumab clearance is entirely consistent with renal physiology, as the large size of nivolumab (146 kDa) is expected to prevent it from being filtered through the renal glomeruli and its elimination via the urine. The pharmacokinetics of nivolumab is linear in the dose range of 0.1 to 10 mg/kg. Body weight normalized dosing produced approximately uniform steady-state trough concentration over a wide range of body weights (34-162 kg). In phase I-II clinical trials, the antitumour activity with respect to objective response rates approached a plateau at 3 mg/kg, with no increased benefit observed at doses of >3 mg/kg [15]. Therefore, the dose of 3 mg/kg was chosen for the phase III clinical trials such as the CheckMate-025 trial for patients with mRCC. In September 2016, based on simulations by the population pharmacokinetics model, the US FDA modified the recommended dosage regimen of nivolumab (for RCC, metastatic melanoma, and non-small cell lung cancer) from 3 mg/kg to a flat dose of 240 mg IV every two weeks. Although the metabolic pathway of nivolumab has not been fully characterized, it is expected to be degraded into small peptides and amino acids via catabolic pathways in the same manner as endogenous IgG.

The current evidence base for using nivolumab in patients with ESRD and dialysis is limited to the following three case reports. Carlo* et al*. reported the case of a 77-year-old male with mRCC and ESRD on dialysis who received 4^th^-line treatment with nivolumab [4]. Although the patient developed respiratory failure after 1 dose of nivolumab, he appeared to have a partial response subsequently and remained on nivolumab treatment for 8 months. Another case of metastatic clear cell RCC and ESRD on dialysis that responded to nivolumab was reported by Tabei and colleagues recently [5]. Ong et al. reported the case of a 63-year-old female with postrenal transplant metastatic cutaneous melanoma, where treatment with 1 dose of nivolumab led to acute renal allograft rejection and renal failure, subsequently requiring dialysis [6]. Rechallenge with nivolumab in this patient achieved a radiological response for over 8 months.

Our case report adds further to literature by highlighting the usage of nivolumab in an elderly patient with mRCC with ESRD and dialysis. Despite multiple comorbidities, he tolerated the treatment well with minimal treatment-related toxicities. Treatment with nivolumab was associated with a partial radiological response as per RECIST v1.1 along with an improvement in pain control and performance status. Although the median duration of nivolumab treatment in the CheckMate-025 trial was 5.5 months, it is interesting to note that, despite the above-mentioned comorbidities, our patient has been on nivolumab treatment for 22 months with ongoing treatment response.

## 4. Conclusions

In a resource-limited healthcare setting, clinicians may opt to strictly follow the inclusion criteria of the pivotal trials and show reluctance to offer nivolumab to mRCC patients with ESRD on dialysis. However, the evidence outlined in this article demonstrates the pharmacokinetic data and case studies to make a compelling case for the consideration of nivolumab in patients with mRCC and ESRD requiring dialysis after failure of prior antiangiogenic therapy. We recommend that patients with mRCC with ESRD and dialysis should be treated with standard protocols applicable to those with normal renal function.
